# Transcriptome revealed the molecular mechanism of *Glycyrrhiza inflata* root to maintain growth and development, absorb and distribute ions under salt stress

**DOI:** 10.1186/s12870-021-03342-6

**Published:** 2021-12-16

**Authors:** Ying Xu, Jia-hui Lu, Jia-de Zhang, Deng-kui Liu, Yue Wang, Qing-dong Niu, Dan-dan Huang

**Affiliations:** 1 College of Life Science, Shihezi University, Shihezi, 832003 Xinjiang China; 2 Licorice Research Institute of Shihezi University, Shihezi, 832003 Xinjiang China; 3 Key Laboratory of Xinjiang Phytomedicine Resource Utilization, Ministry of Education, Shihezi University, Shihezi, 832003 Xinjiang China; 4 Xinjiang Production and Construction Corps Key Laboratory of Oasis Town and Mountain-basin System Ecology, Shihezi University, Shihezi, 832003 Xinjiang China

**Keywords:** *Glycyrrhiza inflata*, Salt tolerance, RNA-Seq, Transcriptome analysis, Candidate genes

## Abstract

**Background:**

Soil salinization extensively hampers the growth, yield, and quality of crops worldwide. The most effective strategies to counter this problem are a) development of crop cultivars with high salt tolerance and b) the plantation of salt-tolerant crops. *Glycyrrhiza inflata,* a traditional Chinese medicinal and primitive plant with salt tolerance and economic value, is among the most promising crops for improving saline-alkali wasteland. However, the underlying molecular mechanisms for the adaptive response of *G. inflata* to salinity stress remain largely unknown.

**Result:**

*G. inflata* retained a high concentration of Na^+^ in roots and maintained the absorption of K^+^, Ca^2+^, and Mg^2+^ under 150 mM NaCl induced salt stress. Transcriptomic analysis of *G. inflata* roots at different time points of salt stress (0 min, 30 min, and 24 h) was performed, which resulted in 70.77 Gb of clean data. Compared with the control, we detected 2645 and 574 differentially expressed genes (DEGs) at 30 min and 24 h post-salt-stress induction, respectively. Gene Ontology (GO) and Kyoto Encyclopedia of Genes and Genomes (KEGG) pathway analyses revealed that *G. inflata* response to salt stress post 30 min and 24 h was remarkably distinct. Genes that were differentially expressed at 30 min post-salt stress induction were enriched in signal transduction, secondary metabolite synthesis, and ion transport. However, genes that were differentially expressed at 24 h post-salt-stress induction were enriched in phenylpropane biosynthesis and metabolism, fatty acid metabolism, glycerol metabolism, hormone signal transduction, wax, cutin, and cork biosynthesis. Besides, a total of 334 transcription factors (TFs) were altered in response to 30 min and 24 h of salt stress. Most of these TFs belonged to the MYB, WRKY, AP2-EREBP, C2H2, bHLH, bZIP, and NAC families.

**Conclusion:**

For the first time, this study elucidated the salt tolerance in *G. inflata* at the molecular level, including the activation of signaling pathways and genes that regulate the absorption and distribution of ions and root growth in *G. inflata* under salt stress conditions. These findings enhanced our understanding of the *G. inflata* salt tolerance and provided a theoretical basis for cultivating salt-tolerant crop varieties.

**Supplementary Information:**

The online version contains supplementary material available at 10.1186/s12870-021-03342-6.

## Background

The area under saline-alkaline soil is continuously increasing due to climatic changes and agricultural production [1]. Soil salinity is a severe environmental abiotic stress, which hampers the productivity and quality of crops. It affects about 800 million hectares of land worldwide, including 30% of the world’s highly productive irrigated land [2]. According to a prediction, nearly half of the world’s arable land will be salinized by 2050 [3]. The best strategy to tackle this situation is developing and planting economically valuable salt-tolerant plants in saline-alkaline land [4].

In Chinese Pharmacopoeia (2015 edition), *Glycyrrhiza inflata* is one of the three *Glycyrrhiza* species of medicinal licorice [5]. It is mainly distributed in the Tarim Basin and the salt-alkali desert meadows of the Turpan-Hami basin in the Xinjiang region of China [6]. *G. inflata* thrives in saline-alkali abandoned farmlands. Besides, it positively affects the recovery of saline-alkaline abandoned farmland [7]. Salt tolerance of *G. inflata* is higher than *Glycyrrhiza uralensis* Fisch and *Glycyrrhiza glabra* L. Interestingly, *G. inflata* salt tolerance is much higher than commercial crops and forages of the same family, such as wild soybean and pea [8, 9]. Thus, *G. inflata* has a superior salt tolerance as well as economic and medicinal values. Also, it is one of the most efficient medicinal plants as far as the improvement of saline-alkaline abandoned farmland is concerned. However, the molecular mechanism for *G. inflata* salt tolerance remains largely unknown.

Plant growth, root development, and Na^+^ accumulation in leaves reflect the plant’s salt tolerance under salt stress conditions [10]. Hormonal signal transduction is involved in plant growth regulation and root development under salt stress conditions [11]. For instance, to regulate plant growth, abscisic acid (ABA) signals mediate the transmission of stress signals from root tips and regulate biological processes, such as stomatal conductance [12]. Ethylene reduces the number and elongation of root hairs [13]. The response of plant roots to salt stress can be categorized into four stages: a) stagnation period (root growth rate declines), b) quiescent period (slow root growth), c) recovery period (rapid increase in root growth rate), and d) equilibrium period (stable root growth). ABA signal regulates root growth recovery and lateral root stasis. Gibberellin (GA) and brassinosteroids (BR) signals promote salt stress-induced growth inhibition [11]. Previous studies have shown that 150 mM NaCl treatment for 21 days did not affect *G. inflata* root diameter, root length, and dry weight [9]. However, the underlying hormonal signaling and molecular mechanisms involved in the maintenance of *G. inflata* root growth and development remain elusive.

Storing ions in the roots is an effective way to reduce the sodium ion content in plant leaves [14]. Plants eliminate harmful ions from their roots, primarily at the structural and molecular levels. A vast array of harmful ions are blocked outside the stele by the endothelial barrier at the structural level. Moreover, ion transporters unload or transport harmful ions from the xylem to the extracellular space of plant cells at the cellular level. Thus, these harmful ions are either stored in the roots or excluded from the plants.

The endothelial barrier includes the apoplast barrier, i.e., Casparian strips (CS), and the transcellular barrier, i.e., suberin lamellae. The first stage of endothelial differentiation is the formation of the apoplast barrier. This apoplast barrier blocks the free diffusion of harmful ions into stele [15], which forces stele to pass through the endothelial barrier through either plasmodesmata mediated symplast pathway or ion transporters mediated transcellular pathway [16]. Chemical staining analysis of *Arabidopsis thaliana* showed that the CSs were mainly composed of lignin [17]. In the second stage of endothelial differentiation, suberin is deposited around the endothelial cells on the inner surface of the cell wall [18], inhibiting the transcellular pathway [19]. Suberin, a glycerin-based heteropolymer with a complex chemical structure, comprises aliphatic groups linked to phenolic components [20, 21]. As per previous studies, suberin confers salt tolerance to *Arabidopsis* by inhibiting Na^+^ influx, K^+^ efflux, and the recirculation of water [22].

Our previous studies have shown that under salt stress conditions, differentiation time of endodermis and synthesis of suberin lamella and CSs closer to the root tip are regulated, and Na^+^ into the stele is blocked in *G. inflata* [9]. However, the underlying molecular mechanism remains unknown. Na^+^ is retained in the root or excreted from the plant under salt stress conditions due to the presence of an ion transporter in the roots, which mediates the absorption of essential cations. For instance, salt overly sensitive 1 (SOS1), high-affinity K^+^ transporter (HKT), and sodium/hydrogen antiporter (NHX) are primarily involved in the absorption, long-distance transportation, and distribution of Na^+^ and K^+^ [23]. However, ion transporters that play an active role in the response of *G. inflata* to salt stress remain ambiguous.

In recent years, a plethora of plant transcriptome data has been generated through next-generation sequencing technology. These data have enabled the identification of the molecular mechanisms of different plant species under different stress conditions, extending our current understanding of the molecular mechanisms of plant stress responses, such as *Arachis hypogaea* L. [24], Giant Juncao (*Pennisetum* spp.) [25], *Brassica napus* L. [26], and so on.

In the current study, RNA-seq of the *G. inflata* roots under salt stress at three different time points was obtained by employing the next-generation sequencing technology. This study aimed (1) to investigate the overall differences in the expression levels of *G. inflata* root genes, (2) to reveal the mechanism associated with the molecular response pathway involved in the maintenance of growth and development, and Na^+^ retention and tolerance in *G. inflata* root at different salt stress stages, and (3) to discover the key genes that are involved in retention and tolerance of high concentration of Na^+^ and growth and development of *G. inflata* roots.

## Result

### MDA content in *G. inflata* at different stages of salt stress

**Fig. 1.**
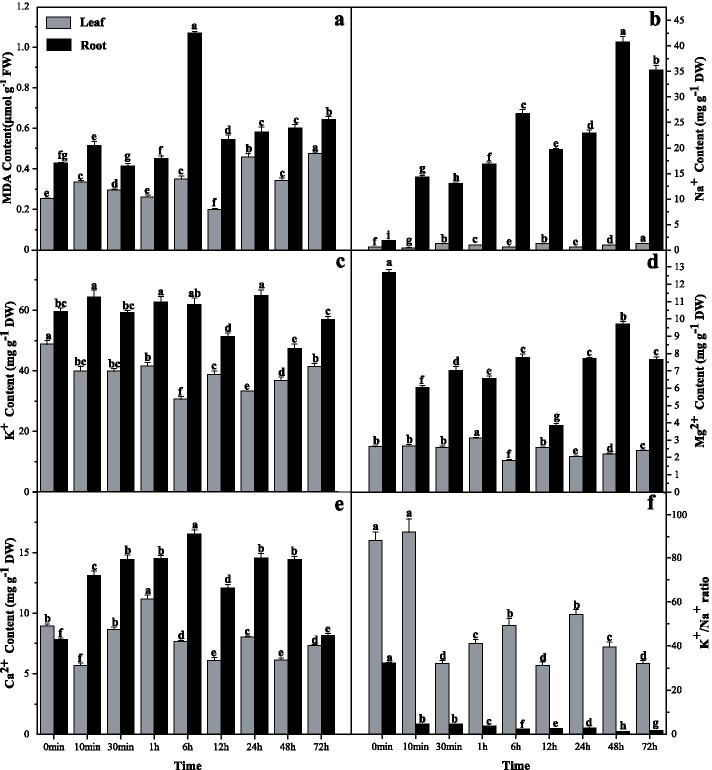
*G. inflata* roots and leaves MDA and ion content at different time points of salt stress. **a** MDA content, **b** Na^+^ content, **c** K^+^ content, **d** Mg2^+^ content, **e** Ca2^+^ content, and **f** K^+^/Na^+^ ratio. 30-days-old *G. inflata* seedlings were precultured in a standard nutrient solution and treated with 150 mM of NaCl for 0 min, 10 min, 30 min, 1 h, 12 h, 24 h, 48 h, and 72 h. For each time point, three replicates were measured independently. Mean ± SE (*n* = 3). Columns marked with the same letters were not significantly different based on the LSD test (*P* < 0.05)

Malondialdehyde (MDA) levels in roots and leaves of *G. inflata* did not increase continuously with increasing salt stress time. At certain time points of salt stress (roots: 10 min ~ 30 min, 6 h ~ 12 h, 24 h ~ 48 h; leaves: 10 min ~ 1 h, 6 h ~ 12 h, 24 h ~ 48 h), the MDA content change was either significantly lower or significantly unaltered compared to control (Fig. 1a). This indicated that the roots and leaves of *G. inflata* could resist or alleviate the oxidative stress induced by salt stress. The MDA content in *G. inflata* roots under salt stress was significantly higher than in leaves at any given time point, indicating that the oxidative damage in roots was higher under salt stress conditions.

### K^+^, Ca^2+^, Na^+^, and Mg^2+^ content in *G. inflata* at the different time points of salt stress

The *G. inflata* roots and leaves Na^+^ content ratio was 10.48 at 30 min and 43.86 at 48 h post-salt-stress induction (Fig. 1b). This indicated that most of the Na^+^ were mainly intercepted by the roots of *G. inflata* under salt stress conditions. This, in turn, significantly increased the K^+^/Na^+^ ratio by 7.05 ~ 34.06 folds in leaves than in root. Although the K^+^ content of leaves was significantly lower than control (Fig. 1c), it did not decline with increasing salt stress at certain time points of salt stress (10 min ~ 30 min, 1 h ~ 6 h, 12 h ~ 24 h). In *G. inflata* roots, the K^+^ content increased significantly at 0 to 10 min, 30 min to 6 h, 12 to 24 h, and 48 to 72 h post-salt-stress induction. At 10 min, 1 h, 6 h, and 24 h, the K^+^ content of roots was significantly higher than the control. The Mg^2+^ level in roots did not decrease continuously with increasing salt stress time; however, it increased significantly at 10 to 30 min, 1 to 6 h, and 12 to 48 h post-salt-stress induction (Fig. 1d). In *G. inflata* leaves, the Mg^2+^ level was not significantly lower than control within 1 h of salt stress, but it increased significantly at 6 to 12 h and 24 to 72 h post-salt-stress induction. The Ca^2+^ content in the leaves showed a waveform change with increasing salt stress time (Fig. 1e). The Ca^2+^ content in the root of *G. inflata* is higher than the control at all time points after salt stress (Fig. 1e). The Ca^2+^ content in roots is highest at 6 h (approximately 2.12 folds of the control), and lowest at 72 h (approximately 1.05 folds of the control) post-salt-stress induction (Fig. 1e). The Ca^2+^ content in leaves is the highest at 1 h (approximately 1.25 folds of the control), and the lowest at 10 min (approximately 0.64 folds of the control) post-salt-stress induction (Fig. 1e). These results indicate that *G. inflata* has the ability to absorb K^+^, Ca^2+^, and Mg^2+^ under salt stress.

### Transcriptomic analysis in the root of *G. inflata* response to salt stress

Transcriptome from the roots of *G. inflata* seedlings at three different salt stress time points was sequenced using Illumina 2000, and a total of nine transcriptome libraries were constructed (three library repeats for each time-point). Low-quality reads and the reads with poly-N were removed, and more than 40 million clean reads (Table S1) were obtained for each sample with Q30 > 92.82% and GC% between 44.49 to 44.74% (Table S2). The sequence output and quality met the set criteria, and sequences were thus further analyzed.

**Fig. 2:**
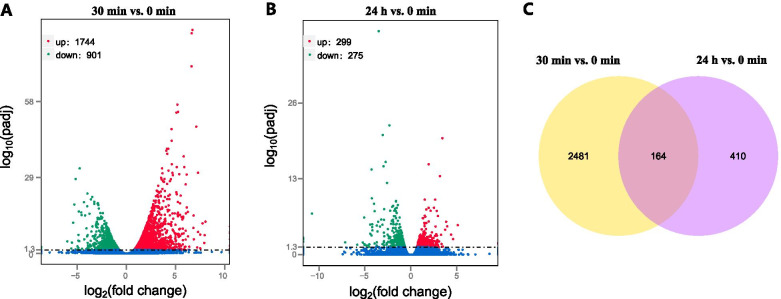
DEGs under salt stress conditions. The DEGs were identified by comparing the gene expression values at different time points (30 min & 24 h) of salt stress with control (0 min). **a** DEGs at 30 min vs. DEGs at 0 min of salt stress, **b** the DEGs at 24 h vs. DEGs at 0 min of salt stress, and **c** Venn analysis of DEGs at 30 min and 24 h post-salt-stress induction compared to control

TopHat software was used to map the clean reads of each sample to the *G. uralensis* Fisch genome (http://ngs-data-archive.psc.riken.jp/Gur-genome/). The proportion of total mapped reads ranged from 67.09 to 69.81% (31,413,445 to 39,260,526 reads) (Table S1). The gene expression levels were calculated and normalized using the TMM method; |log2(FoldChange)| > 1 and adjusted *p*-value < 0.05 were set as the threshold for significant differential expression. A total of 2645 DEGs (1744 up-regulated and 901 down-regulated) and 574 (299 up-regulated and 275 down-regulated) DEGs were detected at 30 min and 24 h post-salt-stress induction, respectively compared to control (Fig. 2). The number of DEGs at 30 min time point was about five times higher than at 24 h time point. Venn analysis was performed to examine the differential expression of root tissue at different time points under salt stress conditions (Fig. 2C). With control as a reference, 164 DEGs intersected at 30 min and 24 h, and 2481 DEGs intersected at 30 min, and 410 DEGs intersected at 24 h post-salt-stress induction. To validate the transcriptome data, expression levels of eight transcripts were determined using quantitative real-time PCR, and the outcomes were compared with RNA-seq data. We obtained a positive correlation coefficient (*R*^*2*^ = 0.86914) for these eight transcripts using linear regression analysis, which indicates the reliability of RNA-seq data (Fig. S1).

#### Gene Ontology (GO) analysis of *G. inflata* DEGs under salt stress conditions

**Fig. 3:**
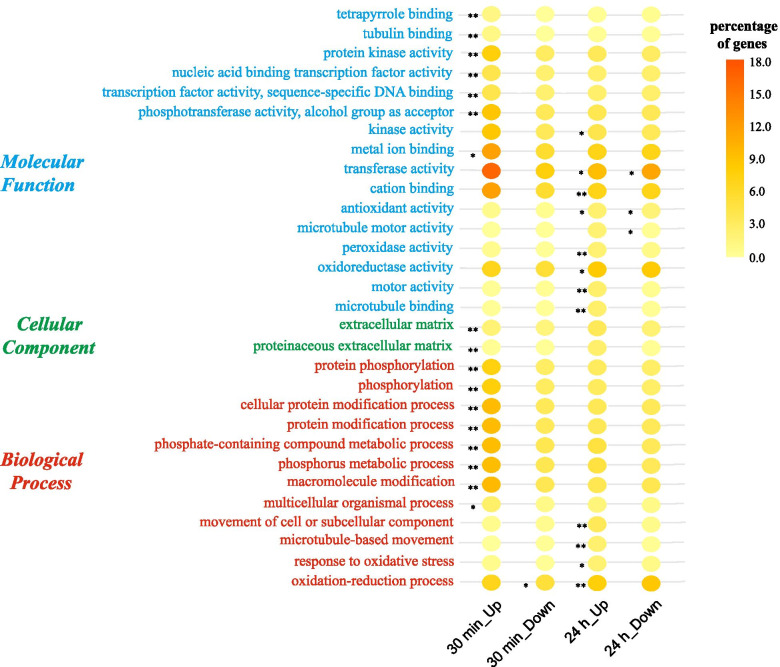
GO analysis of DEGs under 30 min and 24 h salt stress. All of the GO terms at level 2 were shown. The bubble diagrams are GO terms overrepresented among up-and down-regulated genes in *G. inflata* roots at different time points under salt stress conditions. The darker the color bubbles, the higher the proportion of up- or down-regulated genes in the GO term. “*” indicates significant enrichment (*P* < 0.05); “**” indicates extremely significant enrichment (*P* < 0.01)

To explore the functional significance of DEGs, up-and down-regulated DEGs from two different time points in salt stress conditions were subjected to GO analysis (Fig. 3). A total of 17 GO terms including “protein kinase activity,” “metal ion binding,” “nucleic acid binding transcription factor activity,” “extracellular matrix,” “macromolecule modification,” and so on, were mainly enriched in the upregulated DEGs at 30 min post-salt stress induction. The DEGs down-regulated at 30 min of salt stress were only significantly enriched in the “oxidation-reduction process.” Furthermore, a total of 12 GO terms including “oxidation-reduction process,” “response to oxidative stress,” “movement of a cell or subcellular component,” “antioxidant activity,” “kinase activity,” and so on, were mainly enriched in the upregulated DEGs at 24 h post-salt stress induction. The DEGs down-regulated at 24 h of salt stress were significantly enriched in “microtubule motor activity,” “antioxidant activity,” and “transferase activity.”

#### Kyoto Encyclopedia of Genes and Genomes (KEGG) pathway enrichment analysis of *G. inflata* DEGs under salt stress conditions

**Fig. 4:**
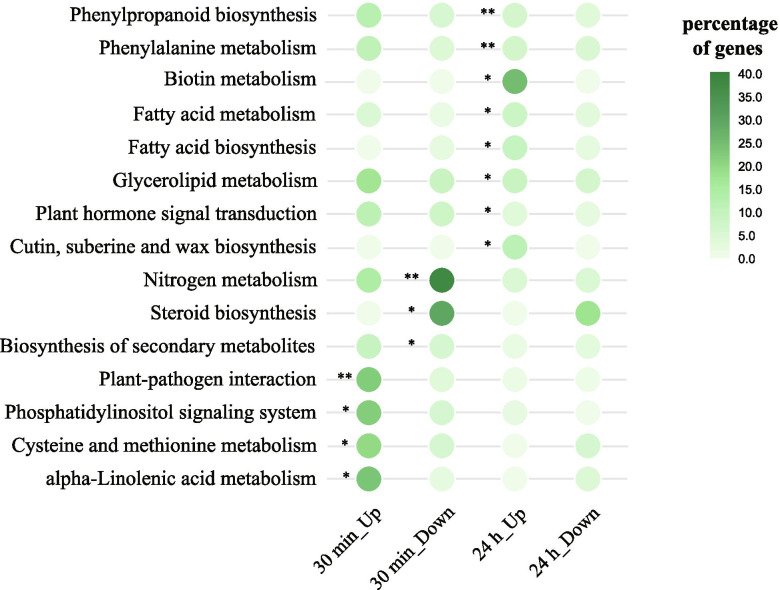
The KEGG pathway enrichment analysis. The KEGG representation analyses. The bubble diagrams are KEGG pathways overrepresented between up and down-regulated genes in *G. inflata* roots under different salt conditions. The darker the color bubbles, the higher the proportion of up- or down-regulated genes in the KEGG pathways. “*” means significant enrichment (*P* < 0.05); “**” means extremely significant enrichment (*P* < 0.01)

To unravel the activation of biological pathways in *G. inflata* in response to salt stress, the KEGG pathway enrichment analysis of DEGs was performed. The results showed enrichment of metabolism and signal transduction pathways. The DEGs that were up-regulated at 30 min post-salt-stress induction were enriched in 89 biological pathways. Out of these 89 pathways, 4 signaling pathways, namely, plant-pathogen interaction, phosphatidylinositol signaling system, cysteine, and methionine metabolism, and alpha-linolenic acid metabolism, were significantly enriched. The down-regulated DEGs at 30 min post-salt-stress induction were enriched in 79 pathways. Out of these79 pathways, three pathways, namely, nitrogen metabolism, steroid biosynthesis, and biosynthesis of secondary metabolites were significantly enriched. The up-regulated DEGs at 24 h post-salt-stress induction were enriched in 47 biological pathways. Out of these 47 pathways, eight pathways, namely, phenylpropanoid biosynthesis, phenylalanine metabolism, biotin metabolism, fatty acid metabolism, fatty acid biosynthesis, glycerolipid metabolism, plant hormone signal transduction, and cutin, suberin, and wax biosynthesis were significantly enriched. The down-regulated DEGs from 24 h post-salt-stress induction were enriched in 68 biological pathways, but none of the pathways were significantly enriched (Fig. 4). Enrichment of these pathways might indicate their activation under salt stress conditions. Also, DEGs at 30 min and 24 h post-salt-stress induction were not enriched for a common biological pathway. This indicated that the salt response process of *G. inflata* is dynamic and has different response strategies to salt stress at different time points.

##### Activation of hormonal signal transduction in *G. inflata* roots in response to salt stress

**Fig. 5:**
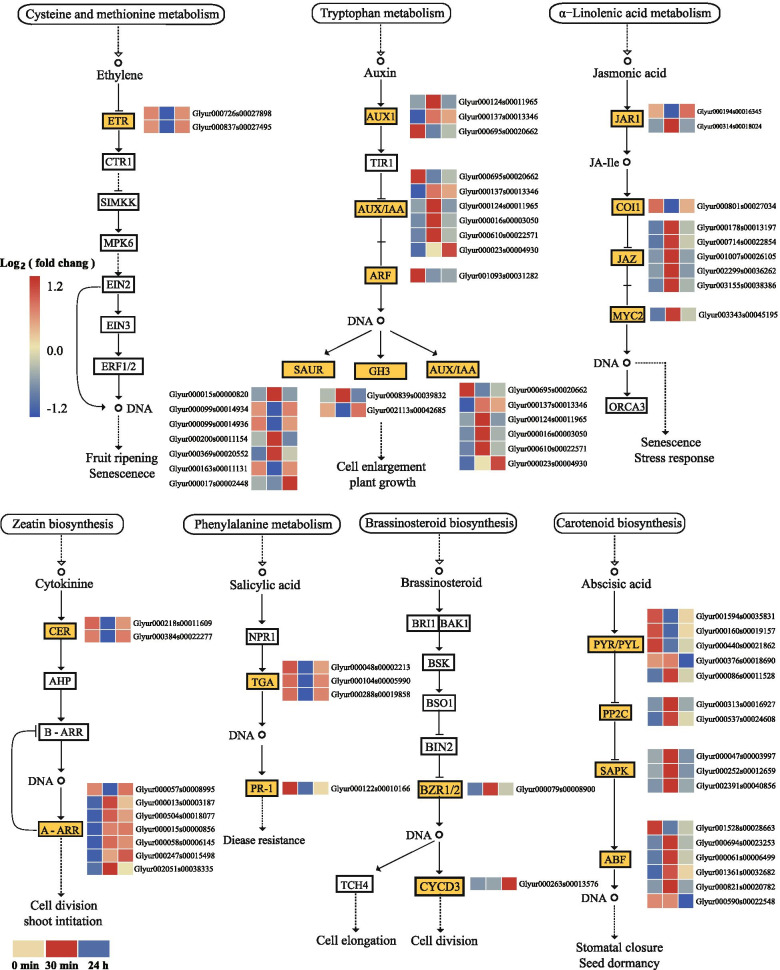
Heat map of DEGs involved in plant hormone signal transduction pathways. The relative expression levels of DEGs were calculated using the log2 ratio

Phytohormones are involved in the regulation of plant growth and other biological processes as well as adaptation to stress conditions. In this study, we performed KEGG pathway enrichment analysis for DEGs expressed under salt stress conditions. The results suggested that salt stress induces activation of various hormonal signaling pathways, such as auxin, cytokinin, ABA, ethylene, jasmonic acid, salicylic acid, and so on in *G. inflata* root under salt stress (Fig. 5). Multiple genes related to ABA, auxin, and jasmonic acid signal transduction, were differentially expressed at 30 min, but there was no significant change at 24 h post-salt-stress induction. These hormonal signaling may play an important role in the early response of *G. inflata* to salt stress. All DEGs involved in ethylene and salicylic acid signal transduction were down-regulated at 30 min and did not show significant change at 24 h post-salt-stress induction. Thus, we speculated that ethylene and salicylic acid signaling were inhibited at the initial stage of salt stress. The cyclin-D3-1 (CYCD3) coding gene expression, a response factor involved in BR signal transduction, did not change significantly at 30 min, and it was up-regulated at 24 h post-salt-stress induction. We speculate that BR signaling may play a crucial role at 24 h post-salt-stress induction. The cytokinin receptor *CER1* coding gene was down-regulated at 30 min, and it did not change significantly at 24 h post-salt-stress induction. However, the expression pattern of the cytokinin response factor A-ARR encoding gene was not consistent with the *CER1* expression, but the A-ARR encoding gene was up-regulated at 30 min and 24 h post-salt-stress induction.

##### The biosynthesis of secondary metabolites in *G. inflata* in response to salt stress

In KEGG pathway enrichment analysis, 83 up-regulated and 52 down-regulated DEGs at 30 min post-salt-stress induction were enriched in the biosynthesis of secondary metabolites. Besides, 14 up-regulated and 25 down-regulated DEGs at 24 h post-salt-stress induction were enriched in the biosynthesis of the secondary metabolite pathway (Table S3). These DEGs were found to be involved in the synthesis of a variety of secondary metabolites, such as phenylpropanoids, suberin, hormones, steroids, flavonoids, and triterpenoids (Table S3).

**Fig. 6:**
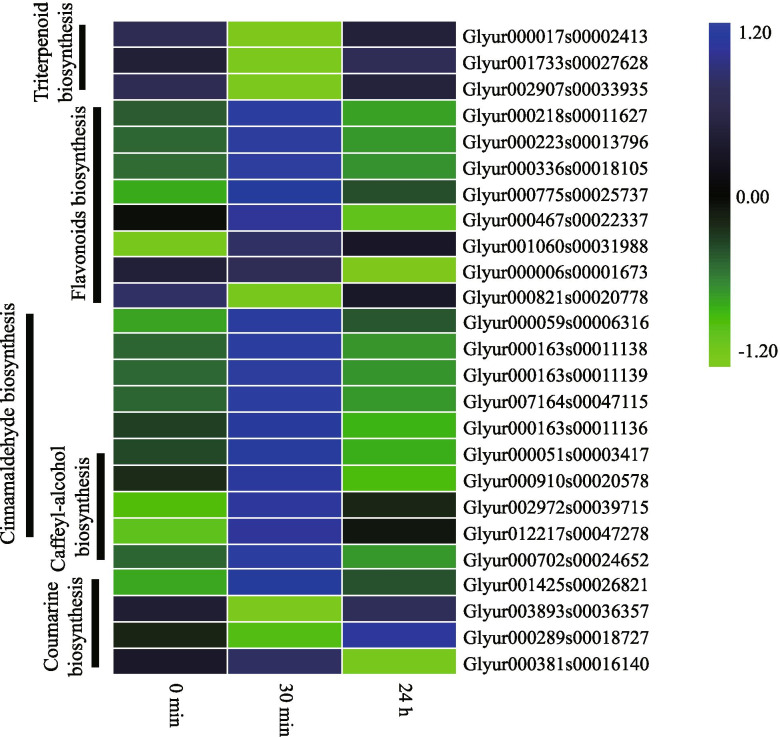
DEGs involved in the biosynthesis of some secondary metabolites, including triterpenoid, flavonoids, caffeoyl-alcohol, cinnamaldehyde, and coumarin. The relative expression level of genes was calculated using the log2 ratio

DEGs enriched in the triterpenoid biosynthesis pathway were down-regulated at 30 min and had no significant differential expression at 24 h post-salt-stress induction (Fig. 6). Out of these DEGs, the Glyur000017s00002413 gene encoding squalene synthase plays a key role in triterpenoids biosynthesis. This indicates that triterpenoids biosynthesis in *G. inflata* was inhibited during the initial period of salt stress and reverted to a normal state at 24 h post-salt-stress induction. We speculate that the inhibitory effect of 150 mM NaCl salt stress on the triterpenoid biosynthesis in *G. inflata* is temporary.

The majority of the DEGs involved in the flavonoid biosynthesis were up-regulated at 30 min and had no significant change at 24 h post-salt-stress induction (Fig. 6). Thus, we speculated that short-term salt stress stimulates the flavonoids biosynthesis in *G. inflata*.

A total of 37 DEGs were found to be involved in phenylpropanoid biosynthesis and metabolism. These DEGs were also involved in the biosynthesis of lignin, caffeoyl-alcohol, cinnamaldehyde, and coumarin. Out of these 37 DEGs, 4 beta-glucosidase (BGL) protein-encoding DEGs were related to coumarin biosynthesis. DEGs related to the biosynthesis of caffeoyl-alcohol and cinnamaldehyde were up-regulated at 30 min and did not change significantly at 24 h post-salt-stress induction (Fig. 6). This indicated that the biosynthesis of caffeoyl-alcohol and cinnamaldehyde was activated in the early stage of salt stress. At 30 min post-salt-stress, we found that 11 up-regulated DEGs are related to the biosynthesis of lignin monomers. A total of 22 DEGs were identified as peroxidases (POX), involved in the catalysis of the lignin synthesis from the lignin monomer. Out of these 22 DEGs, 7 DEGs were up-regulated at 30 min, and 7 DEGs were up-regulated at 24 h post-salt-stress induction. A total of 3 DEGs involved in the cutin, suberin, and wax biosynthesis pathway, were up-regulated only at 24 h post-salt-stress induction. This indicates activation of the biosynthesis of cutin suberin and wax 24 h after post-salt-stress induction.

### Activation of other biological pathways in *G. inflata* in response to salt stress

A majority of the genes involved in the interaction pathway between plants and pathogens were up-regulated at 30 min post-salt-stress induction. These genes include calcium-dependent protein kinase genes, disease-resistant protein genes, calcium-binding protein genes, WRKY transcription factors, and so on (Fig. S2). This indicates that this pathway is activated at the initial stage of salt stress and may transmit external environmental information in *G. inflata*. Thus, we speculated that it might play a vital role in the early adaptation of *G. inflata* to salt stress.

In KEGG pathway analysis, 14 DEGs were enriched in the phosphatidylinositol signaling system. Out of these 14 DEGs, 11 DEGs were up-regulated at 30 min, and one DEG was up-regulated at 24 h post-salt-stress induction. This indicated that the enriched pathways were mainly activated at the early stage of salt stress (Fig. S3). Multiple studies have shown that these pathways are involved in signal transduction in response to stress, but the exact function of each element in this pathway remains largely unknown.

A total of 5 DEGs were enriched in fatty acid biosynthesis pathways, of which four genes were up-regulated only at 24 h post-salt-stress induction, and one gene was down-regulated at both 30 min and 24 h post-salt-stress induction. These genes were involved in the biosynthesis of long-chain fatty acids. This indicates that the long-chain fatty acid biosynthesis was more active at 24 h post-salt-stress induction (Table S4). Out of the total 16 DEGs enriched in glycerolipid metabolic pathway (Table S5), three DEGs (Glyur000006s004755, Glyur000370s00222656, and Glyur001572s00035827) encoding glycerol-3-phosphate acyltransferase were up-regulated only at 24 h post-salt-stress induction.

### Transcription factors (TFs) in *G. inflata* in response to salt stress

**Fig. 7:**
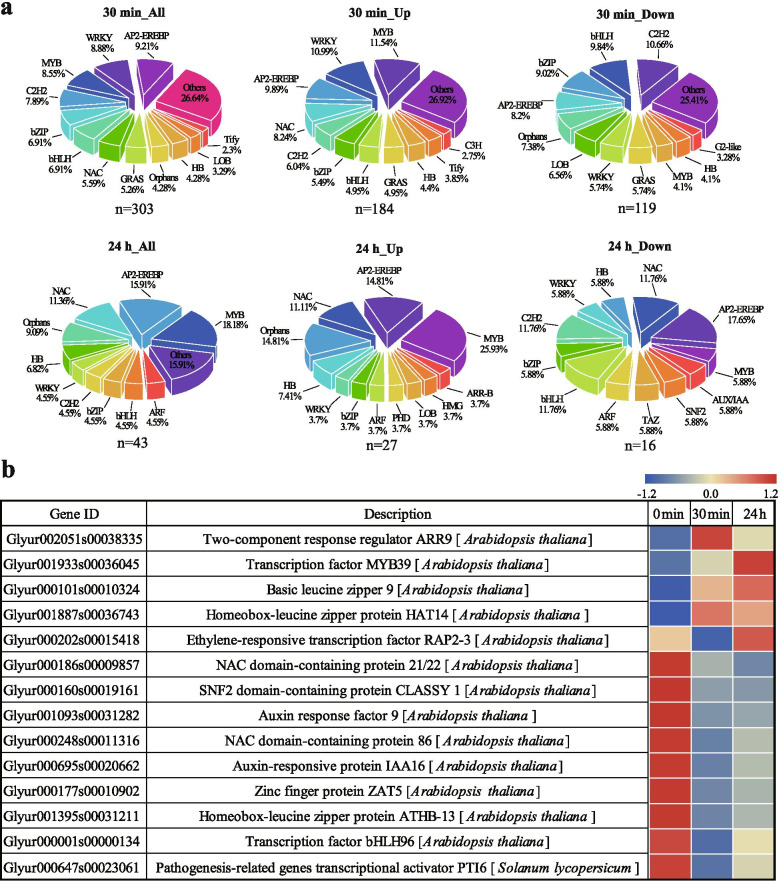
Transcription factors (TFs) differential expression in response to salt stress in *G. inflata*. **a** Statistics of up-regulated and down-regulated TFs induced by salt stress. **b** Differentially expressed TFs at 30 min and 24 h post-salt-stress induction

We found that a total of 334 DEGs were enriched as TFs. Most of these TFs belonged to the MYB, WRKY, AP2-EREBP, C2H2, bHLH, bZIP, and NAC families (Fig. 7a). Out of these 334 DEGs, 290 TFs were differentially expressed (178 up-regulated and 112 down-regulated) at 30 min, and 30 TFs were differentially expressed (22 up-regulated and 8 down-regulated) at 24 h post-salt-stress induction. Besides, 14 TFs were differentially expressed (4 up-regulated and 9 down-regulated) at 30 min and 24 h post-salt-stress induction, and one TF were up-regulated at 30 min and down-regulated at 24 h post-salt-stress induction (Fig. 7b). We speculate that these 14 continuously differentially expressed TFs may play a crucial role in the response to salt stress of *G. inflata.*

### Ion transporters in *G. inflata* in response to salt stress

**Fig. 8:**
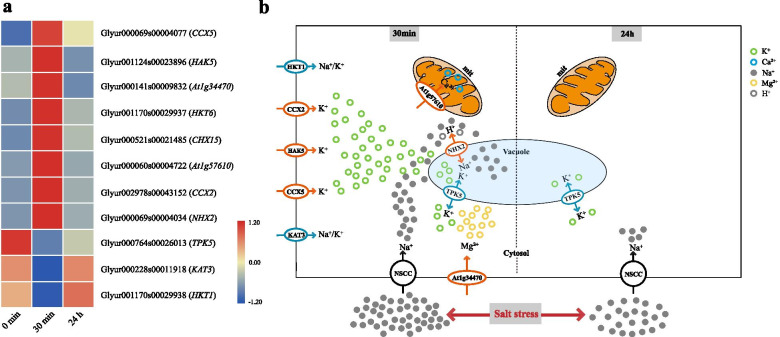
Differentially expressed genes encoding ion transporters. **a** The expressions of the possible ions transporters or channels identified in the transcriptome. **b** Ion transport models at different salt stress time points

Transporters play a crucial role in the Na^+^ absorption, transport, and chelation. We analyzed DEGs encoding ion transporters. After 30 min of salt induction, NHX2, HAK5, CCX5, CCX2, CHX15, HKT6, Atlg57610, and Atlg34470 were up-regulated, and HKT1, KAT3, and two-pore potassium channel 5 (TPK5) were down-regulated. Surprisingly, only TPK5 was down-regulated at 24 h post-salt-stress induction. This suggests that ion transporter primarily responds at the initial stage of salt stress (Fig. 8a).

## Discussion

### *G. inflata* salt tolerance

During the early stage of salt stress, the plant’s water absorption capacity is reduced due to concomitant osmotic stress, resulting in ionic toxicity [27]. The core mechanism of salt stress response involves the transport of water and ions (mainly Na^+^, Cl^−^, and K^+^) between the plant and environment, as well as their redistribution between different plant parts and cellular compartments [28]. In this study, the Na^+^ content in the *G. inflata* root was much higher than that in the leaves. This shows that most of the Na^+^ absorbed from the environment were retained in the roots of *G. inflata*. Salt-tolerant plant species or genotypes commonly demonstrate a low Na^+^ accumulation in shoots and a high Na^+^ accumulation in roots under salt stress conditions [29]. In halophytes or salt-tolerant plants, absorption, transportation, and separation of inorganic ions as osmotic regulators are cost-effective methods to counter osmotic stress [30, 31]. Due to low Na^+^ accumulation in the leaves of *G. inflata*, the minimum K^+^/Na^+^ ratio is 32.06. A potassium-to-sodium ratio of about 1.0 is essential to maintain the steadystate of ions [32]. In *G. inflata*, the interception of Na^+^ in the roots might be involved in the maintenance of a high K^+^/Na^+^ ratio and reduced ion toxicity in the shoots, and increased osmotic stress in the roots caused due to salt stress. However, overcoming the effects of response to salt stress is a highly challenging task for the cells in the *G. inflata* roots. High Na^+^ accumulation decreases the level of other essential metal cations, such as Ca^2+^ and Mg^2+^, which in turn affects physiological and biochemical plant activities [33]. In this study, the levels of K^+^, Ca^2+^, and Mg^2+^ in *G. inflata* roots and leaves increased significantly at some stages of salt stress. This indicates that *G. inflata* can resist or alleviate the inhibitory effect of salt on the absorption of other essential metal cations, thereby maintaining normal growth and development. In summary, *G. inflata* has strong salt tolerance; it can maintain the absorption of essential cations under salt stress and retain Na^+^ in the roots.

It is worth mentioning that in addition to acting as an essential nutrient element for plants, Ca^2+^ also plays an important role in the process of information transmission [34]. With the increase of the salt stress time, the Ca^2+^ content in the roots and leaves of *G. inflata* shows a waveform change (Fig. 1e). Plants possess a rapid stress signaling system based on Ca^2+^ waves [35], which regulates various Ca^2+^-dependent enzymes and pathways to resist salt stress [36]. We speculate that the fluctuating changes of Ca^2+^ content in *G. inflata* under salt stress is the result of its comprehensive regulation in order to meet the needs of growth, development, and information transmission. The Ca^2+^ content in the root of *G. inflata* during 48 h ~ 72 h post-salt-stress induction decreased significantly from 1.844 times of the control to 1.05 times of the control (Fig. 1e). This is similar to the research results of *Medicago sativa L.* [37], *Salicornia brachiate* [38], *Chloris virgata* [39], etc. Abiotic stress can stimulate the efflux of Ca^2+^ in plants at certain moments [40]. The significant and continuous increase of Ca^2+^ results in irreversible damage to the cells [41]. We speculate that the significant decrease of Ca^2+^ content in 48 ~ 72 h of salt stress may represent a self-protection measure of *G. inflata* or the weakening of calcium signal. The physiological mechanism and significance involved in this process need to be further studied.

In addition to ionic and osmotic components, salt stress can also give rise to oxidative stress by increasing the levels of reactive oxygen species (ROS) [42]. MDA is the byproduct of damaged cell membrane caused due to higher ROS content than the threshold [43, 44]. Generally speaking, the longer the salt stress time, the higher is the MDA content. However, in the current study, the MDA content of *G. inflata* showed fluctuations and did not increase continuously with the increasing salt stress time; specifically, the MDA content in the roots of *G. inflata* decreased from 2.67 folds of the control to 1.36 folds during 6-12 h of salt stress (Fig. 1a). A large number of studies have shown that the plant antioxidant system can effectively remove ROS and reduce the damage caused by oxidative stress [45]. We speculate that *G. inflata* can activate the antioxidant system under salt stress conditions, alleviate lipid peroxidation caused due to oxidative stress.

### Regulation of ion transporter-mediated absorption and compartmentalization of ions in *G. inflata* roots under salt stress conditions

The Glyur0000069s000004034 encoding NHX2 was up-regulated 30 min post-salt-stress induction (Fig. 8a). The protein encoded by the *AtNHX2* gene separates the Na^+^ from the cytoplasm and store it in the vacuoles to prevent toxicity [46]. As Na^+^ and K^+^ have a similar ionic radius, certain K^+^ transporters can mediate Na^+^ absorption under salt stress conditions. In the current study, compared with the control, the Glyur001170s00029938 encoding HKT1 in *G. inflata* was down-regulated at 30 min post-salt-stress induction, and no significant change was observed 24 h post-salt-stress induction (Fig. 8a). These findings were in line with the previous study on salt tolerance in *Gossypium hirsutum* seedlings [47]. Kader et al. reported that the OsHKT1 down-regulation in salt-tolerant leaf mesophyll cells was due to hampered Na^+^ transport in these metabolically active cells [48]. Thus, we speculate that *NHX2* up-regulation and *HKT1* down-regulation lead to the distribution of Na^+^ to the vacuole and alleviation of Na^+^ influx in *G. inflata*.

The Glyur001124s00023896 and Glyur000069s00004077 genes encoding HAK5 and CCX5, respectively, participate in K^+^ transport. These genes were differentially up-regulated at 30 min post-salt-stress induction, and their expression levels did not change significantly at 24 h post-salt-stress induction (Fig. 8a). CCX5 and HAK5 can mediate K^+^ absorption under low potassium and salt stress conditions [49, 50]. Moreover, the *OsHAK5* gene knockout mutant showed a reduced K^+^/Na^+^ ratio in the shoot, indicating that *OsHAK5* may be involved in K^+^ distribution between roots and shoots [51]. In the current study, the K^+^ level in *G. inflata* leaves was significantly higher at 30 min post-salt-stress than at 24 h post-salt-stress induction (Fig. 1c). This suggests that the Glyur001124s00023896 gene participated in long-distance K^+^ transportation at 30 min post-salt-stress induction.

Glyur001170s00029937 and Glyur002978s00043152 genes encoding HKT6 and CCX2 were also differentially up-regulated 30 min post-salt-stress induction (Fig. 8a). However, no detailed study is available on the function and location of these genes. Glyur002978s00043152 gene, which encodes TPK5 protein, was down-regulated 30 min post-salt-stress induction (Fig. 8a). TPK5, a putative entity, encodes voltage-independent potassium channels that are located in the vacuoles. Five similar putative entities have been identified in *A. thaliana*, namely TPK1, TPK2, TPK3, TPK4, and KCO3. However, so far, only TPK1 and TPK4 have been explored [52]. We speculate that in *G. inflata,* vacuolar two-pore potassium channel activity is regulated through the *TPK5* down-regulation. This, in turn, reduces the K^+^ transport from the cytoplasm into the vacuole and increases the storage space in the vacuole for the Na^+^, allowing K^+^ participation in various biological processes, such as enzyme activation and protein synthesis in the cytoplasm.

Glyur000060s00004722 gene, which encodes mitochondrial calcium uniporter (MCU), was differentially up-regulated at 30 min post-salt-stress induction (Fig. 8a). MCU5, a selective calcium channel located in the inner membrane of mitochondria, mediates Ca^2+^ influx [53] and plays a crucial role in calcium homeostasis in mitochondria and cells [54].

Interestingly, only one gene encoding TPK5 was down-regulated 24 h post-salt-stress induction (Fig. 8b), suggesting a weak response of ion transporter to salt stress. Compared with 30 min of salt stress, the K^+^, Na^+^, and Mg^2+^ levels at 24 h increased significantly, and Ca^2+^ levels did not change significantly. K^+^, Ca^2+^, Na^+^, and Mg^2+^ levels at 24 h increased significantly compared to 12 h of salt stress; however, the K^+^/Na^+^ ratio did not change significantly (Fig. 1). These results indicate that although the regulation of ion transporters was not significant at 24 h post-salt-stress induction, uptake of K^+^, Ca^2+^, and Mg^2+^ and basic ion homeostasis was maintained. We speculate that before 24 h of salt stress, *G. inflata* established new ions and osmotic pressure dynamic balance, mitigating the need for increased ion absorption and efflux.

### Sequestration of Na^+^ into *G. inflata* roots through CSs development under salt stress conditions

**Fig. 9:**
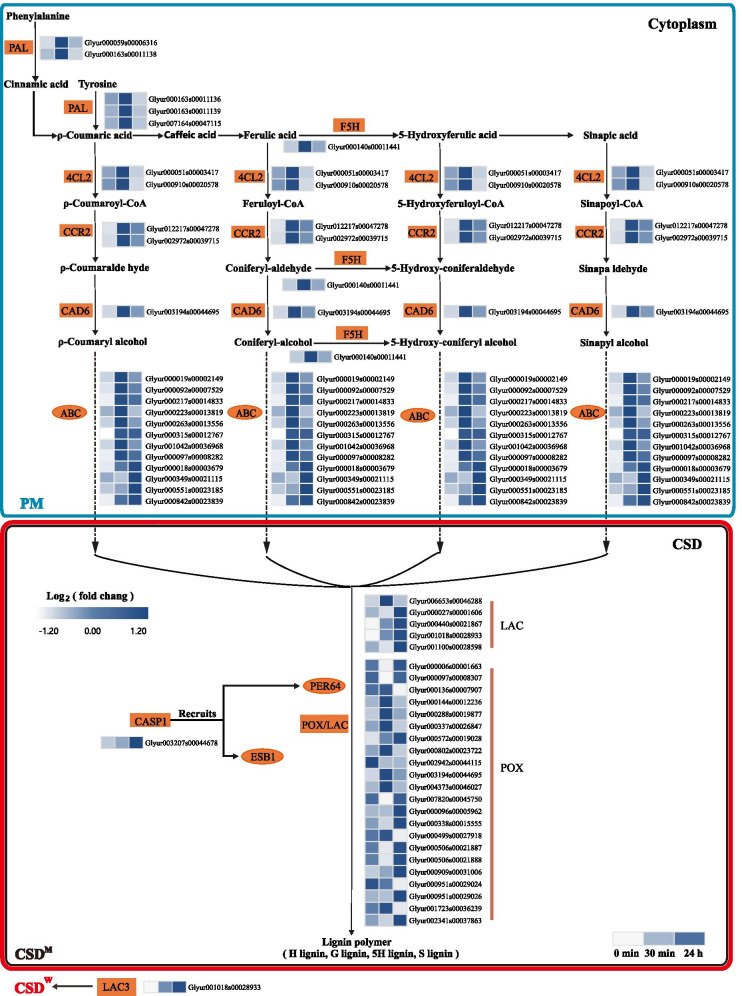
Heat map of differentially expressed genes involved in lignin biosynthesis and Casparian strip formation under the different stages of salt stress

The CSs acts as a physical barrier in the apoplast transport [55] and hinder the ion flow into the stele [56], and promote the plant response to different environmental stress conditions [57]. In the current study, we observed that most of the genes related to the biosynthesis of lignin, a crucial component of the CSs, were differentially up-regulated under salt stress conditions. Out of these lignin synthesis genes, *PAL1*, *PAL3*, *PALY*, *4CL2*, *CCR2*, *CAD6*, and *CYP84A1* genes related to lignin monomer biosynthesis were differentially up-regulated at 30 min post-salt-stress induction. These genes catalyze the synthesis of ρ-Coumaric acid, ferulic acid, 5-Hydroxyferulic acid, and sinapic acid into S-type, H-type, 5H, and G-type lignin monomer, respectively (Fig. 9). PAL catalyzed reaction is one of the rate-limiting steps in lignin synthesis. Also, increased expression and activity of PAL mostly signifies the environmental stress in plant tissues [58]. CYP84A1 gene encodes ferulate-5-hydroxylase (F5H). In comt deletion mutants, *CYP84A1* overexpression resulted in the accumulation of 5H type suberin monomers; *CYP84A1* overexpression resulted in S-type lignin monomers to account for 90% of the total lignin monomers [59, 60]. We speculate that salt stress could stimulate *G. inflata* to produce H-type, S-type, G-type, and 5H-type lignin monomers 30 min post-salt-stress induction.

As the lignin monomer synthesis does not occur directly in the cell wall but in the cytoplasm or near the endoplasmic reticulum [58], a series of transport mechanisms are required to make the lignin monomer pass through the cell membrane to reach the cell wall. The ABC transporter can be used to transport lignin monomers [61]. In the current study, we observed that a total of 12 genes encoding ABC transporters are differentially up-regulated. Thus, we speculate that these genes might be involved in the transport of lignin monomers in response to salt stress in *G. inflata*.

The genes encoding LAC and POX were up-regulated at 30 min and 24 h post-salt-stress induction. The lignin monomers were oxidized and polymerized into lignin under the action of plant laccases (LAC) [62] and type III peroxidases (POX) [63]. The *CASP1* and *LAC3* were up-regulated at 24 h post-salt-stress induction, but their expression did not change significantly at 30 min post-salt-stress induction. As the cellular location of LAC, POX, and lignin varies, the site of lignin formation in the cell also varies. The condition for the CS formation is the lignin deposition in the formation area of the CS. CASP1 recruits peroxidase and proteins, such as PER64 and ESB1, related to lignin formation to accumulate in the formation area of the CS [61]. Thus, regulation of the *CASP1* expression is essential for the CS membrane domain (CSD^M^) construction and CS formation [64]. Recent studies have reported the LAC3 regulated CS wall domain (CSD^W^) is spatially separated from CSD^M^ by CASP1. Besides, CSD^W^ works in coordination with CSD^M^ for the precise lignification of CS [65]. We speculate that *G. inflata* responds to salt stress by forming CS at 24 h post-salt-stress induction.

In summary, *G. inflata* could respond to salt stress through lignin synthesis, increasing cell integrity, mechanical support, and water transport [66]. At 24 h post -salt stress, in response to salt stress, CS formation could prevent Na^+^ from entering the stele [56], thus responding to salt stress.

### Sequestration of Na^+^ into *G. inflata* roots through suberin lamellae development under salt stress conditions

Suberin entails aliphatic, phenolic, and glycerin monomers [20], which are deposited around the endothelial cells on the inner surface of the cell wall to form suberin lamellae, blocking the transport of water and solutes through transcellular pathways. Thus, suberin is vital for conferring abiotic stress tolerance, such as salinity and drought in plants [18]. At 24 h post-salt-stress induction, the up-regulated genes involved in phenylpropanoids, fatty acids, glycerides, and suberin biosynthesis, were significantly enriched in most biological pathways related to the biosynthesis and assembly of suberin monomers (Fig. 4). Thus, we speculate that at 24 h post-salt-stress induction, suberin biosynthesis increases in *G. inflata* in response to salt stress.

**Fig. 10:**
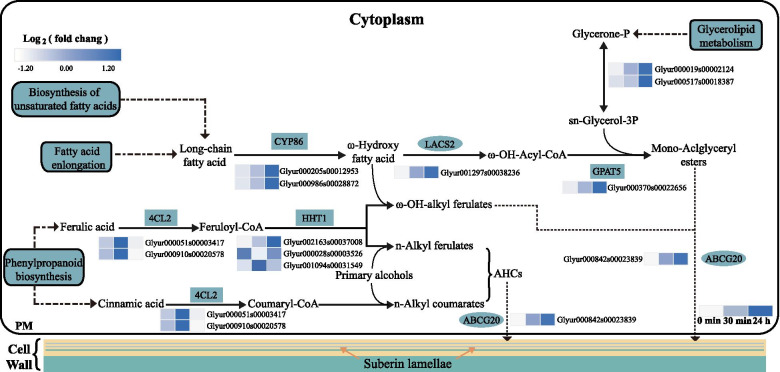
Heat map of differentially expressed genes involved in suberin lamellae formation under different salt stress stages

**Fig. 11:**
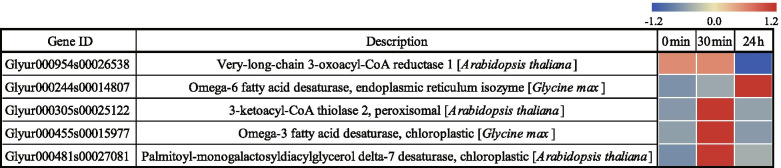
Heat map of DEGs involved in the unsaturated fatty acid pathway. Relative expression was calculated by log2 ratio

The aliphatic monomers of suberin include ω-hydroxy fatty acids, α, ω-dicarboxylic acids, unsaturated fatty acids, medium-chain oxygenated fatty acids, and primary fatty alcohols [20, 64]. In the current study, we observed that salt stress stimulates the biosynthesis of aliphatic monomers ω-hydroxy fatty acids and unsaturated fatty acids. The genes encoding CYP86A1 and CYP86B1 proteins were up-regulated at 24 h post-salt-stress induction (Fig. 10). These enzymes promote the ω-hydroxy fatty acid formation, of which CYP86A1 catalyzes the formation of short-chain ω-hydroxy fatty acids from C12 to C18 fatty acids, which is particularly important in the early deposition of primary endodermis suberin [67]. CYP86B1 catalyzes the formation of long-chain omega-hydroxy fatty acids from C22 to C24 fatty acids [68]. We speculate that the synthesis of short-chain and long-chain omega-hydroxy fatty acids in *G. inflata* is more active at 24 h post-salt-stress induction compared to control. The differential expression of genes encoding unsaturated lipases (FAD), which regulate fatty acid unsaturation, were up-regulated at 30 min and 24 h post-salt-stress induction (Fig. 11). The *FAD7* was also up-regulated in barley roots under salt stress conditions [69]. We speculate *FAD7* and *FAD2-2* overexpression in *G. inflata* under salt stress conditions to enable increased synthesis of unsaturated fatty acids and suberin for countering the salt stress.

*GPAT5* encoding GPATs proteins were up-regulated 24 h post-salt-stress induction. GPATs are primarily involved in transferring the acyl group of fatty acid acyl-CoA to the sn-2 position of glycerol triphosphate to produce monoacylglycerol [70]. Monoacylglycerol is the original suberin component [16]. Previous studies have shown that suberin level in roots of *gpat5* mutant *Arabidopsis* was reduced by 50%; however, the composition and content of membranes, stored glycerolipids, and surface waxes remain unchanged [71]. Also, long-chain acyl-CoA synthetase 2 gene *LACS2* was up-regulated at 24 h post-salt-stress induction. LACS2 protein plays a crucial role in the ω-hydroxyacyl-COA synthesis [72]. However, the involvement of this gene in the biosynthesis of suberin aliphatic monomers remains elusive. We believe that LACS2 is involved in converting suberin aliphatic monomer, ω-hydroxy fatty acids are converted into ω-hydroxyacyl-CoA under salt stress conditions. The main glycerol monomer of suberin is glycerol-3-phosphate (G-3-P). According to a current study, G-3-P biosynthesis in plants depends on glycerol kinases (GKs) [73], glycerol triphosphate dehydrogenase [74], and α-glycerophosphate oxidase [75]. In the current study, we observed that the two genes encoding p-glycerol-3-phosphate dehydrogenase [NAD^(+)^] 1 were up-regulated at 24 h post-salt-stress induction. The genes encoding this protein were also reported in the rice genome [76]. We speculated this gene might be involved in the synthesis of G-3-P under salt stress conditions. The phenolic monomers of suberin primarily include alkyl hydroxycinnamate (AHC), ferulic acid, coumaric acid, and lignin monomers [77]. Out of these phenolic monomers, the biosynthesis of lignin monomer and coumaric acid was found to be more active at 30 min post-salt-stress induction (Fig. 9). Ferulic acid generates ferulic acid acyl-CoA in a 4CL catalyzed reaction followed by transfer of acyl groups to ω-hydroxy fatty acids and fatty alcohols in an ASFT/HHT catalyzed reaction to generate ω-OH-alky-ferulates and n-Alky-ferulates [68]. In our study, the genes encoding 4CL were up-regulated at 30 min of salt stress, and the genes encoding HHT1 were up-regulated at 30 min and 24 h of salt stress (Fig. 10). This indicates that salt stress stimulates the AHCs synthesis in *G. inflata*.

The suberin precursors synthesized in *G. inflata* under salt treatment, including monomers or partially formed oligomers, are transported from the endoplasmic reticulum to the plasma membrane where suberin lamellae are formed in the apoplast [21]. Thus, suberin transport is the key step to the formation of the suberin lamellae. In *Arabidopsis*, *abcg2*, *abcg6*, and *abcg20* triple mutant were characterized by the suberin’s altered structure, composition, and properties in the root and seed coat [78]. In the current study, Glyur000842s0023839, which encodes ABCG20 protein, was up-regulated 24 h post-salt-stress induction. This suggested that the Glyur000842s0023839 gene might be involved in the transport of monoacylglycerol, unsaturated fatty acid, alkyl hydroxycinnamate, coumaric acid, and lignin monomer and other suberin precursors from the cytoplasm to the cell wall for suberin lamellae formation.

In summary, the suberin phenolic monomers synthesis is more active at 30 min, and the synthesis of suberin and suberin aliphatic monomers and glycerin monomers is more active at 24 h min post-salt-stress induction. ABCG20, which might be involved in the transport of suberin to apoplasts, is up-regulated at 24 h under salt stress. We speculate that *G. inflata* may respond to salt stress through suberin lamellae formation at 24 h and 30 min post-salt-stress induction.

### Hormonal signaling regulates root growth and development in *G. inflata* under salt stress conditions

Salt tolerance is defined as the plant’s ability to maintain its growth under saline stress [79]. Roots are the only plant part that directly encounters salt stress [80]. The growth and development of most plant roots are inhibited under salt stress conditions, making it difficult for plants to grow in a saline environment. Thus, the *G. inflata* root’s ability to maintain growth and development under salt stress is the key to the strength of plant salt tolerance [78]. Under salt stress, plant hormones primarily regulate root growth and development, which requires tissue or cell-specific signaling networks [81]. In this study, we observed that auxin and abscisic acid signal transduction genes were mainly differentially expressed at 30 min post-salt-stress induction. However, ethylene, jasmonic acid, and salicylic acid signal transduction pathways were only differentially expressed at 30 min post-salt-stress induction. BR and cytokinin signal transduction pathways were continuously activated at 30 min and 24 h min post-salt-stress induction.

Roots dynamically regulate growth and development in response to salt stress, which is restored after a period of transient growth inhibition [11]. Abscisic acid plays a crucial role in regulating lateral root growth and development under salt stress conditions [82]. The genes in this pathway encoding serine/threonine-protein kinase SAPK2 (SAPK2), serine/threonine-protein kinase SAPK3 (SAPK3), and serine/threonine-protein kinase SAPK7 (SAPK7) were all differentially up-regulated only at 30 min of salt stress (Fig. 5). SAPK7 and SAPK2 were expressed in the roots of seedlings [83]. SAPK2 and SAPK3 can phosphorylate BZIP46 along with ABI5, PP2C30, and PYL5 to regulate the growth and development of rice under drought stress conditions [84]. We speculate that SAPK2, SAPK3, and SAPK7 may positively affect the growth and development of the *G. inflata* roots under salt stress conditions.

BR regulates root meristem size [85]. In our study, Glyur000079s00008900, the gene encoding the transcription factor BES1/BZR1 homolog protein 2 (BZR1/2), was up-regulated at 30 min post-salt-stress induction. The gene Glyur000263s00013576, which encodes CYCD3, was up-regulated at 24 h of salt stress induction. CYCD3 activity is crucial in determining cell number in developing lateral organs and the relative contribution of the alternative processes of cell production and cell expansion to overall organ growth [86]. Previous studies have shown that double mutants of cytokinin receptors, *ahk2* and *ahk3*, form a root system with stronger absorptive capacity by increasing the growth rate of the primary root and lateral roots [87]. In this study, Glyur000218s00011609 gene, which encodes histidine kinase 2 (AHK2), and Glyur000384s00022277 gene, which encodes histidine kinase 3 (AHK3), was down-regulated at 30 min post-salt-stress induction. The regulation of plant root growth and development is a complex process. Different hormone signals have different effects on plant growth and development, and there is crosstalk between different hormones [88]. Thus, the plant root growth and development under salt stress conditions and its detailed physiological indicators demand an in-depth investigation.

## Conclusion

In the current study, we obtained 70.77 Gb clean data from RNA-Seq analysis of *G. inflata* roots and identified 2645 and 574 DEGs at 30 min and 24 h post-salt-stress induction, respectively. Most of the DEGs were found to be related to metabolism and signal transduction. A total of 334 TFs were differentially expressed in *G. inflata*. These findings indicate that DEGs involved in salt stress tolerance have a highly significant and complex role. In addition, the differential expression of genes related to ion transporters (*NHX2*, *CCX5*, *HAK5*, *HKT1*, *HKT6*, *TPK5*, *MCU*, *KAT3*), phenylpropanoid biosynthesis and metabolism (*PAL1*, *PAL3*, *4CL2*, *CCR2*, *CAD6*, *CYP84A1*), cutin, suberin, and wax biosynthesis (*CYP86A1*, *CYP86B1*, *HHT1*), fatty acid biosynthesis and metabolism, glycerolipid biosynthesis (*GPAT5*), and endothelial barrier formation (*CASP1*, *LAC3*, *ABC20*) were involved in the regulation of the absorption and distribution of ions in the *G. inflata* cells and organ levels. Furthermore, certain differentially expressed genes related to plant hormone signal transduction (*SAPK2*, *SAPK3*, *SAPK7*, *CYCD3*, *AHK2*, *AHK3*) were involved in the regulation of root growth and development of *G. inflata* under salt stress conditions. This study is the first to explain the salt tolerance of *G. inflata* at the molecular level. To a certain extent, the data from this study explain the underlying molecular mechanisms of salt tolerance in *G. inflata*.

## Materials and methods

### Plant materials and salt treatment

*G. inflata* seeds were procured from Licorice Research Institute, Shihezi University. These seeds were treated with 85% concentrated H_2_SO_4_ for 30 min, immersed in 0.1% HgCl_2_ solution for 10 min, and rinsed 3-5 times with sterile water. Later these seeds were placed in a petri dish containing a moist filter paper and allowed to germinate at 25 °C in an illumination incubator (GXZ-430D) under dark conditions. 7-day-old seedlings with uniform size were transferred to a culture flask filled with containing modified Hoagland nutrient solution. The plants were placed under long-day (14 h light/10 h dark cycle) conditions at a temperature of 28 °C (light) and 22 °C (dark) with irradiation intensity ranging from 280 ~ 420 μmol·m^− 2^ s^− 1^. To ensure the normal growth of seedlings, the nutrient solution was replaced every 3 days. 30-day-old seedlings were treated with a nutrient solution containing 150 mM·L^− 1^ NaCl. The seedlings were divided into two groups; one group was used for transcriptome sequencing and the other group for physiological analysis. For transcriptome sequencing, after 150 mM·L^− 1^ NaCl treatment for 0 min (control), 30 min, and 24 h, the *G. inflata* root samples were frozen using liquid nitrogen and stored at − 80 °C. Each treatment had three biological replicates. Root and leave samples at 0 min (control), 10 min, 30 min, 1 h, 6 h, 12 h, 24 h, and 72 h were collected for MDA and ion level determination. In this treatment, each treatment had three biological replicates.

### MDA level determination

Malondialdehyde (MDA) level was determined according to the method described by Hodges et al. [89]. 0.5 g of fresh leave and 0.5 g of root samples were homogenized in 4 mL of 0.1% (w/v) trichloroacetic acid (TCA) using a vortex. The homogenate was centrifuged at 15,000×g for 15 min. The resulting 1 mL of the supernatant was mixed with 4 mL of TBA reagent (0.5% of TBA in 20% TCA). This reaction mixture was heated at 90 °C for 20 min in a water bath and then quickly cooled in an ice bath. After centrifuging the samples at 13,000×g for 8 min, the absorbance of the supernatant was measured at 440, 532, and 600 nm. MDA level was calculated using the following formula: MDA (μmol·L^− 1^) = 6.45(D532-D600)-0.56D450.

### Ion concentration determination

The *G. inflata* leave and root samples were first dried at 105 °C for 10 min and then at 80 °C to constant weight using 101-1A drying oven. Later, the dried samples were ground and sieved (30 mesh). The 65% HNO_3_ and 30% H_2_O_2_ (volume ratio 8:3) were added to the 100 mg samples, and the resulting mixture was digested in a microwave digestion instrument (Multiwave 3000, Anton Paar GmbH, Australia). The Na^+^, K^+^, Ca^2+^, and Mg^2+^ levels in the digested solution were determined using an inductively coupled plasma-optical emission spectrometer (Thermo Scientific ICAP 6000 Series, Boston, USA), as per the equipment operation manual.

### RNA extraction and quality determination

Total RNA salt-treated root tissues (0 min, 30 min, and 24 h) were extracted using TRIzol reagent. RNA degradation and contamination were monitored using 1% agarose gel. RNA purity was evaluated using the NanoPhotometer® spectrophotometer (IMPLEN, CA, USA). RNA concentration was measured using Qubit® RNA Assay Kit in Qubit® 2.0 Fluorometer (Life Technologies, CA, USA). RNA integrity was assessed using the RNA Nano 6000 Assay Kit of the Bioanalyzer 2100 system (Agilent Technologies, CA, USA).

### Sequencing library preparation for transcriptome

A total of 3 μg RNA per sample was used as input for the RNA sample preparation. Sequencing libraries were generated using NEBNext® Ultra™ RNA Library Prep Kit for Illumina® (NEB, USA), as per manufacturer’s instruction, and index codes were added to attribute sequences to each sample. Briefly, mRNA was purified from total RNA using poly-T oligo-attached magnetic beads. Fragmentation was carried out using divalent cations under elevated temperature in 5X NEBNext First Strand Synthesis Reaction Buffer. First-strand cDNA synthesis was carried using random hexamer primer and M-MuLV Reverse Transcriptase (RNase H-). Subsequently, second-strand cDNA synthesis was performed using DNA Polymerase RNase H. Overhangs and I were converted into blunt ends using exonuclease/polymerase activities. For hybridization, 3′ ends of DNA fragments were adenylated, and NEBNext Adaptor with hairpin loop structure was ligated. To select 150 ~ 200 bp long cDNA fragments, the library fragments were purified using the AMPure XP system (Beckman Coulter, Beverly, USA). Then, 3 μl USER Enzyme (NEB, USA) was used with size-selected, adaptor-ligated cDNA at 37 °C for 15 min followed by 5 min at 95 °C before initiating PCR. PCR was performed using Phusion High-Fidelity DNA polymerase, Universal PCR primers, and Index (X) Primers. Lastly, PCR products were purified (AMPure XP system), and library quality was assessed on the Agilent Bioanalyzer 2100 system. Libraries were sequenced on an Illumina HiSeq 2000 platform to generate paired-end reads of 125 bp/150 bp long reads.

### Data analysis

Raw data (raw reads) of FastQ format were processed using in-house Perl scripts. In this step, clean data (clean reads) were obtained by removing reads containing adapter. Reads containing ploy-N and low-quality reads from raw data. Q20, Q30 scores, and GC content of the clean data were also calculated. Before downstream analysis, the clean reads in each sample were mapped to the *Glycyrrhiza uralensis* genome database (http://ngs-data-archive.psc.riken.jp/Gur-genome/) using TopHat version 2.0.12.

HTSeq version 0.6.1 was used to count the read numbers mapped to each gene. Then, the FPKM value of each gene was calculated based on the gene length and read counts. Differential expression analysis of two conditions/groups (two biological replicates per condition) was performed using the DESeq R package version 1.18.0. The *P*-values were adjusted using Benjamini and Hochberg’s approach to limit the false discovery rate. Genes with an adjusted *P*-value < 0.05 obtained through DESeq were termed as differentially expressed genes. Differential expression analysis of two conditions was performed using the DEGSeq R package version 1.20.0. The *P*-values were adjusted using the Benjamini & Hochberg method. Corrected *P*-value of 0.005 and log_2_ fold change of 1 was set as the threshold for significantly differential expression.

Gene Ontology (GO) enrichment analysis of differentially expressed genes with corrected gene length bias was performed using the GOseq R package. GO terms with corrected *P*-value < 0.05 were considered significantly enriched. Besides, DEGs were subjected to KEGG pathway enrichment analysis. The statistical enrichment of DEGs was tested using the KOBAS version 2.0 web server, and the corrected *P*-value < 0.05 was considered to be significantly enriched in KEGG.

### Quantitative real-time PCR

To validate the accuracy of the RNA-seq results, Real-Time quantitative PCR (RT-qPCR) analysis was performed. cDNAs were synthesized from RNA extracted from plants before (0 min) and after (30 min, 24 h) the salt stress treatments by using RevertAid™ First Strand cDNA Synthesis Kit (Thermo, USA). RT-qPCR analysis was performed as described previously [90]. *Glycyrrhiza inflata* Bat. 18S rRNA was used as an internal control. The qRT-PCR primers are listed in Table S6.

## Supplementary Information

**Additional file 1.**

**Additional file 2.**

**Additional file 3.**

**Additional file 4.**

**Additional file 5.**

**Additional file 6.**

**Additional file 7.**

**Additional file 8.**

**Additional file 9.**

## Data Availability

The datasets generated and analysed during the current study are included in this article, its supplementary information files and in the [NCBI] repository with Accession: PRJNA707665 & PRJNA604474 [https://www.ncbi.nlm.nih.gov/sra/PRJNA707665 & https://www.ncbi.nlm.nih.gov/sra/PRJNA604474].

## Abbreviations

DEGs: Differentially expressed genes
GO: Gene Ontology
KEGG: Kyoto Encyclopedia of Genes and Genomes
TFs: Transcription factors
ABA: Abscisic acid
GA: Gibberellin
BR: Brassinosteroids
CS: Casparian strips
SOS1: Salt overly sensitive 1
HKT: High-affinity K+ transporter
NHX: Sodium/hydrogen antiporter
MDA: Malondialdehyde
BGL: Beta-glucosidase
POX: Peroxidases
MYB: Myeloblastosis
bHLH: Basic helix-loop-helix
bZIP: Basic leucine zipper
NAC: NAM no apical meristem
NHX: Na^+^/H^+^ exchanger
HAK5: Potassium transporter 5
CCX: Cation/calcium exchanger
KAT3: Potassium channel KAT3
TPK: Two-pore potassium channel
MCU: Mitochondrial calcium uniporter
CS: Casparian strip
PAL: Phenylalanine ammonia lyase
4CL2: 4-coumarate--CoA ligase 2
CCR2: C-C chemokine receptor type 2
F5H: Ferulate-5-hydroxylase
LAC: Laccases
CSDM: CS membrane domain
CSDW: CS wall domain
FAD: Unsaturated lipases
GPAT: Glycerol-3-phosphate acyltransferase
G-3-P: Glycerol-3-phosphate
AHCs: Alkyl hydroxycinnamate
4CL: 4-coumarate-CoA ligase
ABC: ATP-binding cassette
ABCG20: ABC transporter G family member 20
BZR1/2: BES1/BZR1 homolog protein 2
CYCD3: Cyclin-D3-1
RT-qPCR: Real-Time quantitative PCR
