# Supplementary figures and images for: Transcriptome revealed the molecular mechanism of Glycyrrhiza inflata root to maintain growth and development, absorb and distribute ions under salt stress
Source: BMC Plant Biol. 2021 Dec 16;21:599. doi: 10.1186/s12870-021-03342-6 (PMC8675533; doi:10.1186/s12870-021-03342-6)

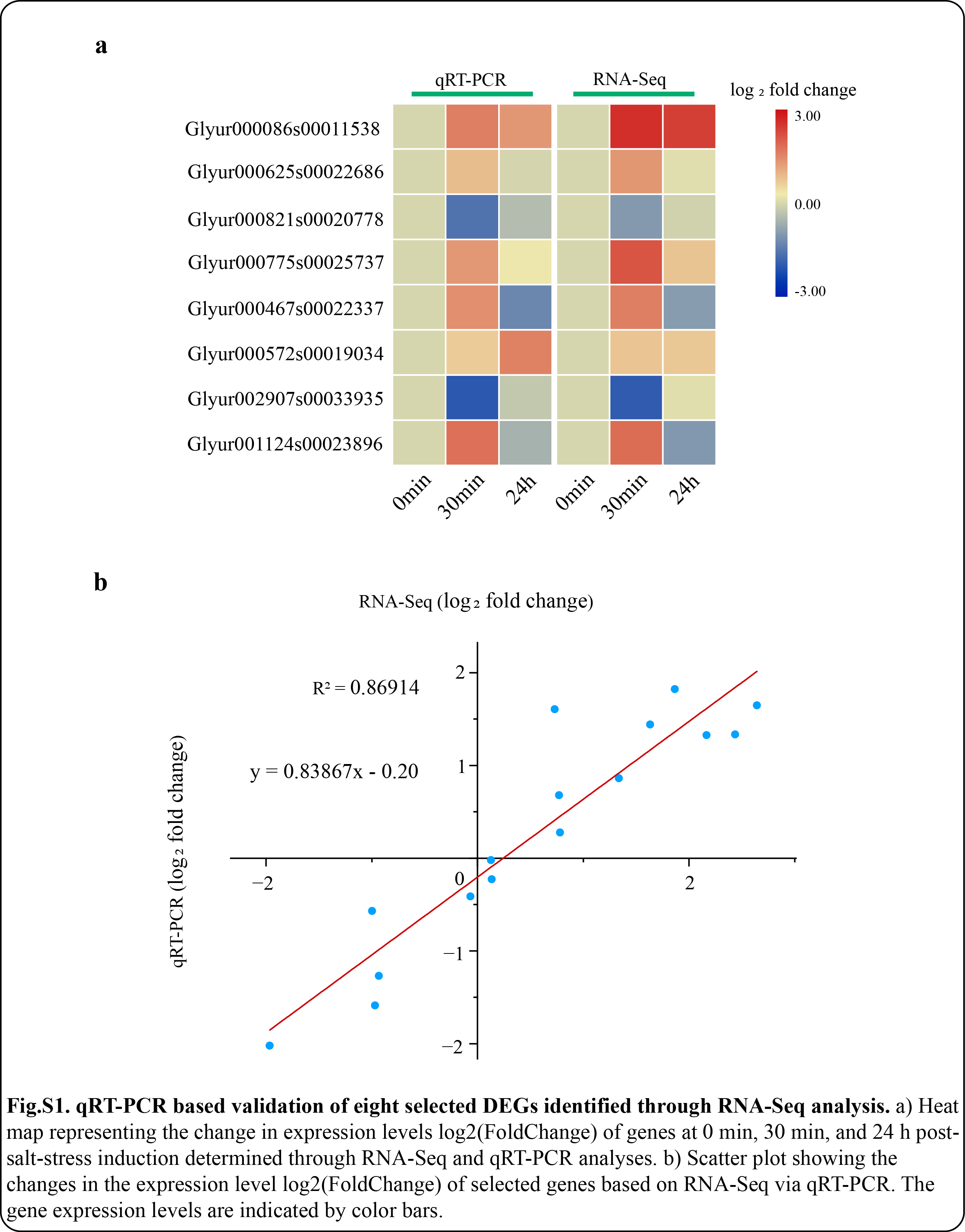

Supplement: Supplementary file 7 — Additional file 7. [file 12870_2021_3342_MOESM7_ESM.png]

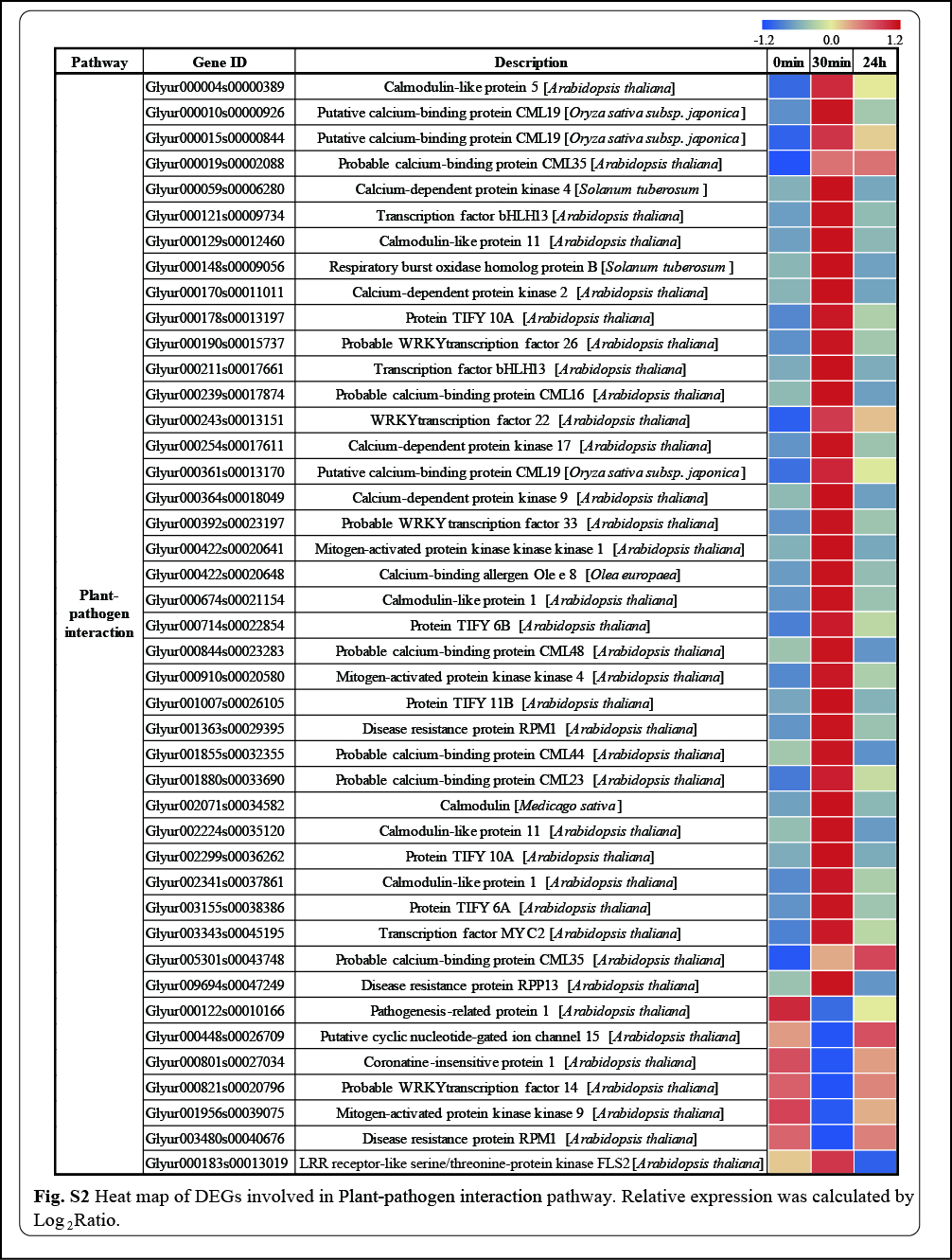

Supplement: Supplementary file 8 — Additional file 8. [file 12870_2021_3342_MOESM8_ESM.jpg]

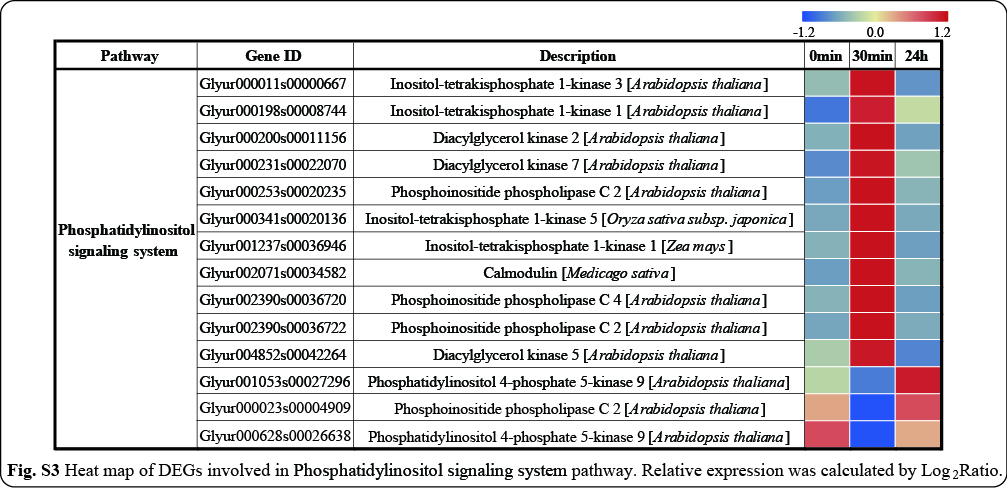

Supplement: Supplementary file 9 — Additional file 9. [file 12870_2021_3342_MOESM9_ESM.jpg]
